# A Novel mRNA-Mediated and MicroRNA-Guided Approach to Specifically Eradicate Drug-Resistant Hepatocellular Carcinoma Cell Lines by Se-Methylselenocysteine

**DOI:** 10.3390/antiox10071094

**Published:** 2021-07-07

**Authors:** Arun Kumar Selvam, Rim Jawad, Roberto Gramignoli, Adnane Achour, Hugh Salter, Mikael Björnstedt

**Affiliations:** 1 Department of Laboratory Medicine, Division of Pathology, Karolinska Institutet, Karolinska University Hospital, S-141 86 Stockholm, Sweden; arun.selvam@ki.se (A.K.S.); rim.jawad@ki.se (R.J.); roberto.gramignoli@ki.se (R.G.); hugh.salter@ki.se (H.S.); 2 Science for Life Laboratory, Department of Medicine Solna, Karolinska Institute, & Division of Infectious Diseases, Karolinska University Hospital, SE-171 77 Solna, Sweden; adnane.achour@ki.se; 3 Moderna, Inc., 200 Technology Square, Cambridge, MA 02139, USA

**Keywords:** Se-methylselenocysteine, kynurenine aminotransferases 1, hepatocellular carcinoma, miRNA, alpha-keto acid, lipid nanoparticle

## Abstract

Despite progress in the treatment of non-visceral malignancies, the prognosis remains poor for malignancies of visceral organs and novel therapeutic approaches are urgently required. We evaluated a novel therapeutic regimen based on treatment with Se-methylselenocysteine (MSC) and concomitant tumor-specific induction of Kynurenine aminotransferase 1 (KYAT1) in hepatocellular carcinoma (HCC) cell lines, using either vector-based and/or lipid nanoparticle-mediated delivery of mRNA. Supplementation of MSC in KYAT1 overexpressed cells resulted in significantly increased cytotoxicity, due to ROS formation, as compared to MSC alone. Furthermore, microRNA antisense-targeted sites for miR122, known to be widely expressed in normal hepatocytes while downregulated in hepatocellular carcinoma, were added to specifically limit cytotoxicity in HCC cells, thereby limiting the off-target effects. KYAT1 expression was significantly reduced in cells with high levels of miR122 supporting the concept of miR-guided induction of tumor-specific cytotoxicity. The addition of alpha-ketoacid favored the production of methylselenol, enhancing the cytotoxic efficacy of MSC in HCC cells, with no effects on primary human hepatocytes. Altogether, the proposed regimen offers great potential to safely and specifically target hepatic tumors that are currently untreatable.

## 1. Introduction

Combinations of first- and second-generation cytostatic drugs, novel targeted therapies including checkpoint inhibitors, multi-kinase inhibitors, and immunotherapies have resulted in remarkable progress in the treatment of non-visceral malignancies, including breast cancer, renal cancer, and malignant melanoma [1,2,3]. However, the prognosis remains poor for visceral malignancies e.g., liver, bile duct, and pancreas cancer, where most efforts so far have led to null or marginal efficacy. Selenium (Se) compounds have been recognized for their cytotoxic properties, particularly in tumor cells resistant to standard cytostatic regimens. The leading cytotoxic mechanism is oxidative stress due to ROS-formation by redox cycles with thiols and oxygen [4]. The therapeutic potential in humans has not been demonstrated yet, due to a lack of tools to increase tumor specificity and insufficient systematic clinical trials.

Methylseleno-cysteine (MSC) is a natural selenium compound produced by plants as a detoxifying agent [5]. MSC may be considered a prodrug since it is inert and non-toxic in the absence of metabolizing enzymes. MSC has high bioavailability in humans [6] and displays favorable pharmacokinetic properties with a short half-life and low risk for chronic selenosis [7]. In mammals, MSC is metabolized primarily by kynurenine aminotransferase1 (KYAT1), a multifunctional, pyridoxal 5′-phosphate (PLP)-dependent enzyme. KYAT1 belongs to a class of enzymes that cleaves carbon-sulfur (C-S) bonds with multiple substrate specificities [8]. This cytosolic enzyme displays dual activities, transamination, and β-elimination on single substrates. The nature of the substrate and the concentration of α-ketoacids determine which pathway will prevail, transamination or β-elimination. KYAT1 has also been shown to effectively cleave C-Se bonds in addition to C-S bonds [9], which renders MSC a suitable substrate for KYAT1. KYAT1 metabolizes the relatively non-toxic MSC through either transamination or β-elimination to β-methylselenopyruvate (MSP) [10] or methylselenol (MS) [11], respectively. Both MS and MSP have profound anti-tumor and anti-proliferative properties [12]. While the tumoricidal activity of MSC has been previously demonstrated in several human cancer cell lines and animal models [13,14,15], MS is one of the most reactive metabolites of selenium and causes oxidative stress with pronounced ROS production and depletion of thiols and NADPH in cells [16,17]. A limited amount of studies indicate that MSP acts as a histone deacetylase (HDAC) inhibitor, leading to increased expression of tumor-suppressor genes [10]. Hence, the selective induction of KYAT1 in tumor cells in the presence of MSC may represent an attractive route to selectively induce cytotoxicity in tumor cells.

RNA-based therapeutics allow the efficient transient induction of proteins via delivery of exogenous mRNA into targeted cells or tissues [18]. The benefit of RNA-based over conventional DNA-based deliveries consists in non-integrating effect into the host genome. Protein translation occurs in the cytoplasm; hence mRNA-based delivery results in rapid and efficient protein expression in tissues [19]. However, the main challenge in RNA-based therapeutics is to selectively induce the protein of interest into the targeted tissues and consequently reduce off-target cell protein expression. MicroRNAs are non-coding small RNA molecules, frequently described as tissue- and disease-specific [20]. Jain et al. demonstrated that the inclusion of microRNA antisense target sites (miRts) into the 3′UTR of mRNAs reduces the expression of introduced mRNA via endogenous microRNA in normal tissues. MicroRNA122 is known to be widely expressed in normal hepatocytes while downregulated in HCC [20]. The liver-specific microRNA miR122 accounts for about 70% of total microRNAs in this organ and is important for liver metabolism and hepatocyte differentiation [21]. The same study proved that miR122 deletion resulted in epithelial-mesenchymal transition (EMT) and spontaneous HCC development in a miR122^−/−^ mouse model [21].

In the present study, we aimed to potentiate MSC cytotoxicity in HCC cell lines by inducing KYAT1 expression via mRNA transfection. We also aimed at mitigating off-target cell protein expression by incorporating microRNA antisense-targeted sites (miRts) specific for normal hepatocytes. Altogether, the presented results indicate that exogenous delivery of LNP-encapsulated KYAT1-encoding mRNA, alongside the pharmacological elevation of MSC levels, may represent a compelling novel strategy to efficiently treat HCC.

## 2. Materials and Methods

### 2.1. Chemicals and Reagents

Potassium dihydrogen phosphate, di-potassium hydrogen phosphate, EDTA, sodium hydroxide, aminooxyacetic acid (AOAA), *L*-tryptophan (*L*-Try), 3-indoleacetic acid (IAA), indole-3-pyruvic acid (IPA), phenylpyruvic acid (PPA), homoserine (HS), *D*L-propargylglycine (PAG), α-keto-γ-(methylthio) butyric acid sodium salt (KMB), Se-methylselenocysteine hydrochloride (MSC), dimethyl-2-oxoglutarate (α-KG), *L*-phenyl alanine (*L*-Phe), 2-amino-2-methyl-1,3-propanediol, pyridoxal 5′-phosphate hydrate (PLP), 2,4-dinitrophenylhydrazine (DNPH), phenylmethanesulfonyl fluoride (PMSF), RIPA buffer, trypan blue, protease inhibitor cocktail mix, *N*-*N*-dimethyl formamide and pLKO.1 vector was purchased from Sigma-Aldrich (St. Louis, MO, USA). BFF-122 was purchased from Axon Medchem (Groningen, The Netherlands), NADPH was purchased from Acros Organics (Geel, Belgium). Lipofectamine 3000 was purchased from Invitrogen (Invitrogen, Camarillo, CA, USA). Page Ruler Plus Prestained protein ladder was purchased from Thermo Fischer Scientific (Rockford, IL, USA). Phusion DNA polymerase, FastDigest Enzymes (EcoRI, KpnI, NotI, XhoI) including buffers, and Rapid DNA Ligation Kit for molecular cloning were purchased from Thermo Fisher Scientific (Rockford, IL, USA). Plasmid pEGFP-N1 (Clontech, Takara Bio Inc, Mountain View, CA, USA) was generously provided by Dr. Gildert Lauter and Dr. Peter Swoboda from the Department for Biosciences and Nutrition, Karolinska Institutet, Stockholm, Sweden. Mammalian TrxR1 was purchased from IMCO (Stockholm, Sweden) and Sigma-Aldrich (Cas no:9074-14-0, Product number T9698, Darmstadt, Germany).

### 2.2. Cell Culture and Growth Condition

HEPG2 cells and Hep3B cells were purchased from ATCC (Wesel, Germany) and Huh7 cells were a generous gift from Dr. Camilla Pramfalk (Division of Clinical Chemistry, Karolinska University Hospital Huddinge, Sweden). These cell lines were maintained in EMEM (ATCC) supplemented with 10% heat-inactivated fetal bovine serum (FBS; Gibco, Paisley, UK), under 5% CO_2_ at 37 °C and without the addition of antibiotics. Cell lines were regularly tested for mycoplasma infection and kept negative. Cell numbers were assessed using a TC 20^TM^ automated cell counter (BioRad, Portland, ME, USA).

Human hepatocytes were isolated as previously described [22] from the surplus surgical waste within the scope of informed consent given by patients undergoing liver resection, and provided by Dr. Ewa Ellis (CLINTEC, Karolinska University Hospital Huddinge, Stockholm, Sweden). The study protocol for the acquisition of cells conformed to the ethical guidelines of the 1975 Declaration of Helsinki and was approved by the Regional Ethics Committee for Human Studies, Stockholm, Sweden, permit number 2017/269-31 and 2019-04866. Human hepatocyte viability was assessed by the trypan blue exclusion method. The retrieved cells were seeded on collagen-treated vessels (at a density of 150,000 viable cells per cm^2^) and maintained in Williams’ medium E (Sigma, Darmstadt, Germany) supplemented with 1 µM dexamethasone, 25 mM HEPES, 12 nM insulin, 2 mM glutamine, and 100 U/mL penicillin and 100 µg/mL streptomycin (Thermo Scientific, Rockwood, TN, USA). 

### 2.3. MSC Cytotoxicity Assessment 

To determine MSC cytotoxicity, cells were seeded in 96-well plates (BD Falcon, Durham, NC, USA) at a density of 400 cells/mm^2^ for HEPG2 and Hep3B cells and 300 cells/mm^2^ for Huh7 cells 24 h before MSC treatment. For the determination of the half-maximal inhibitory concentration (IC_50_), MSC was serially diluted to ten different concentrations. The ATP content of the lysed cells, which is proportional to the number of viable cells, was measured after 48 and 72 h using the luminescence-based CellTiter-Glo^®^ 2.0 cell viability assay kit (Promega, Madison, WI, USA) according to the manufacturer’s instructions with an FLx100 Luminometer (CLARIOSTAR^®^, Ortenbery, Germany).

### 2.4. Cloning of KYAT1 Overexpression Vectors

To obtain a KYAT1 overexpression vector, the KYAT1 wild-type coding sequence was PCR-amplified from cDNA and inserted into the pEGFP-N1 backbone by XhoI and EcoRI restriction digestion. Subsequently, the human PGK promoter and puromycin resistance gene sequences were amplified from pLKO.1 and inserted into the pEGFP-N1 backbone containing the KYAT1 expression cassette by KpnI and NotI digestion. An empty vector was created by inserting the PCR-amplified puromycin resistance gene expression cassette into pEGFP-N1 directly. Thus, the eGFP-coding sequence was replaced with the sequence encoding the puromycin resistance gene in both the KYAT1 overexpression vector and the empty control vector. 

### 2.5. KYAT1 Overexpression via Plasmid

HEPG2 cells were transfected with 2 µg of either KYAT1 overexpression vector or an empty vector using Lipofectamine 3000 (Invitrogen, Camarillo, CA, USA) according to the manufacturer’s protocol. Twenty-four-hour post-transfection, the transfection medium was removed, and the cells were seeded at a density of 400 cells/mm^2^ in 96-well plates for subsequent MSC IC_50_ titration. Successful overexpression of KYAT1 in transfected HEPG2 cells was verified by RT-PCR and Western Blot. 

### 2.6. KYAT1 Overexpression via Formulated (Lipid Nanoparticle Encapsulated) and Unformulated KYAT1mRNA

Complete N1-methylpseudouridine substituted mRNA was synthesized as previously described [23]. After purification, the mRNA was diluted in citrate buffer to the desired concentration and frozen. mRNA sequences of eGFP and KYAT1 were supplied by Moderna Inc. (Cambridge, MA, USA). KYAT1 mRNA with pseudouridine incorporating a 3× miR122 antisense site in the same relative locations within the 3′ UTR as described by Ruchi et al. [20] was purchased from TriLink Biotechnologies (San Diego, CA, USA). These were designated as either unformulated mRNA or formulated mRNA where the latter constructs were encapsulated within specially designed lipid nanoparticles (LNP) as described previously [24]. All formulations were confirmed to be between 80 nm and 100 nm particle size, greater than 80% of RNA encapsulation, and <10 EU/mL endotoxin. For transfection, cells were seeded at a density of 400 cells/mm^2^ or 300 cells/mm^2^ in 60 mm dishes (Sarstedt, North Rhine-Westphalia, Germany) and allowed to grow to 80–90% confluency. Formulated LNP was added at the desired concentration in µg/mL directly to the cell culture media, whereas unformulated mRNA was combined with the respective amount of Lipofectamine 3000 reagent according to the manufacturer’s protocol. Transfected cells were incubated for 4 h followed by washing with PBS with the subsequent addition of fresh medium. Cells were grown for an additional 20 h before they were harvested for protein isolation. To study the protein expression levels over time, cells were transfected with 0.5 µg/mL LNP particles as described above. Transfected cells were harvested at 24, 48, 72, 96, and 120 h post-transfection. To study the protein saturation effects cells were transfected with varying concentrations of KYAT1 ranging from 0.1 to 1 µg/mL and harvested at 48 h post-transfection. These cell lysates were subjected to Western blot for protein quantification and transamination assay for enzyme activity. For the determination of IC_50_ value, cells were further treated as described above under the MSC cytotoxicity section. 

### 2.7. Quantitative Real-Time PCR

Transfected cells were harvested after 24 and 48 h post-transfection. Total RNA was isolated using the RNeasy Plus Mini kit (Qiagen, Hilden, Germany) according to the manufacturer’s instructions. RNA content was measured using a NanoDrop spectrophotometer ND-1000 with subsequent cDNA synthesis using the Ominscript RT kit (Qiagen, Hilden, Germany) according to the provided protocol. RT-PCR was performed using the iQ SYBR green supermix (Bio RAD, Portland, ME). Expression analysis was executed in triplicates and the expression of HPRT was used as a reference gene. PCR primers (Gene accession number: NM_004059, NM_000194) are as follows: 

***KYAT1*** F: CACGCTGTCAGTGGAGACTT, R: TATCTCCTGACCCAGCAGCT.

***HPRT1*** F: GCAGACTTTGCTTTCCTTGG, R: TATCCAACACTTCGTGGGGT.

For microRNA expression analysis, 10 ng of RNA was used for synthesizing cDNA using a Taqman advanced microRNA cDNA synthesis kit (Applied Biosystems Inc., Foster City, CA, USA) followed by Taqman advanced microRNA assay (Applied Biosystems Inc., Foster City, CA, USA) and qRT-PCR was performed by TaqMan fast advanced master mix (Applied Biosystems Inc., Foster City, CA, USA). miR122-5p (#477855-mir) expression analysis was executed in triplicate and the expression of miR192-5p (#478262-mir) and miR16-5p (#477860-mir) was used as reference genes. All miRNAs probes were bought from Applied Biosystems Inc., Foster City, CA, USA.

### 2.8. Quantification Assay for Total Protein Content

Samples were lysed in RIPA buffer (Sigma, Darmstadt, Germany) in the presence of 1 mM phenylmethanesulfonyl fluoride (PMSF) and 1% protease inhibitor cocktail mix (Sigma, Darmstadt, Germany) or 100 mM potassium phosphate EDTA buffer pH 8.0 (for intracellular thiol determination). Further, lysed cells were sonicated at 4 °C for 30 s with 1- to 2-s pulses. The proteins were harvested by centrifugation at 13,000 rpm for 10 min at 4 °C, the supernatant was collected, and protein concentration was determined by the Pierce™ Bicinchoninic acid (BCA) Protein Assay Kit (Thermo Fischer Scientific, Rockford, IL, USA) according to the manufacturer’s instructions.

### 2.9. Western Blot Analysis 

Separation of 20 µg or 50 µg protein from cell lysate was performed on a 10% Mini-PROTEAN^®^ TGX^TM^ gel (BioRad, Portland, ME, USA) and subsequently transferred onto Immunoblot PVDF 0.45 µm membranes (BioRad, Portland, ME, USA) by semi-dry transfer. The membranes were blocked in 5% milk in 1× TBST and immunoblotted with primary antibodies against KYAT1 (1:1000, anti-CCBL1, #C4622) (Sigma, Darmstadt, Germany) overnight at 4 °C. Vinculin (#V284) (1:5000, Millipore, Billerica, MA, USA) was used as a loading control. Membranes were washed and incubated with secondary infrared fluorescent IRDye^®^ antibody (LI-COR^®^, 1:10,000) (LI-COR, Lincoln, NE, USA). Subsequently, membranes were washed three times with 1× TBST, and blot images were acquired in an Odyssey Fc (LI-COR^®^) (Lincoln, NE, USA) Imaging system. Quantification of the protein levels was determined in the Odyssey Image software (LI-COR^®^) (Nebraska, USA) by normalizing the fluorescence intensity of detected KYAT1 antibody signals to the loading control vinculin signal.

### 2.10. Measurement of Intracellular Thiol 

Total intracellular thiol was measured by the DTNB method [25]. Briefly, 10 µL of cell lysates were added to 100 µL of buffer containing 6 M guanidine-HCl, 1 mM DTNB in 0.2 M Tris-HCl, pH 8.0 in a 96-well UV transparent plate (Corning, ME, USA; Cat # 27617048) for 5 min in room temperature. Absorbance was measured at 412 nm in a spectrophotometer (CLARIOSTAR^®^, Ortenbery, Germany). The assay mixture without cell lysate was used as a blank. Under the test condition, the molar extinction coefficient for DTNB was 13,600 M^−1^ cm^−1^.

### 2.11. Measurement of Reactive Oxygen Species and Lipid Peroxidation 

Intracellular ROS was measured spectrophotometrically in cells treated with MSC using H_2_DCFDA and sulforhodamine B protocol described by Neville and Lezanne [26]. Lipid peroxidation was determined in MSC-treated cells using MDA (malondialdehyde) assay kit (colorimetric) according to the manufacturer’s protocol (Abcam, Cambridge, UK, Cat #ab233471). 

### 2.12. KYAT1 Enzyme Activity Assay

KYAT1 transamination activity was measured with whole-cell lysate according to the published procedure [27]. 

Coupled β-elimination activity assay was measured with whole-cell lysate according to the published procedure [28]. To assess the inhibitory effects of transamination and β-elimination enzyme activity, the test compounds were added at the desired concentration as indicated with KYAT1 overexpressed whole cell lysate (20 µg). 

### 2.13. Statistical Analysis

Results are expressed as mean ± SD and represented in Box and Whisker plots or Violin plots showing median, 25- and 75-percentiles. The analysis was performed by one-way ANOVA with 95% confidential interval followed by Tukey’s or Dunnett’s multiple comparison test. Statistical differences between IC_50_ values were determined by fitting nonlinear regression slopes on independent experiments (*n* ≥ 3) and subjecting the data to one-way ANOVA followed by Tukey’s multiple comparison test. Data were analyzed with GraphPad Prism software, version 8.3.3 (GraphPad Software Inc., San Diego, CA, USA).

## 3. Results

### 3.1. Growth Inhibitory Effect of MSC in HCC Cell Lines and Normal Primary Hepatocytes

**Figure 1:**
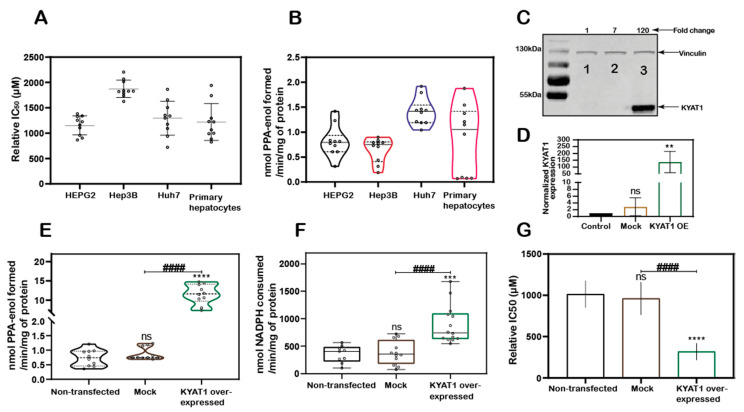
KYAT1 expression and MSC cytotoxicity in cancer and normal human liver hepatocytes. (**A**) MSC cytotoxicity in three different hepatocellular carcinoma cell lines (HEPG2, Hep3B, and Huh7) and freshly isolated human primary hepatocytes at 72 h (*n* = 10). (**B**) Transamination activity of KYAT1 in three HCC cell lines and human primary hepatocyte (*n* = 10). (**C**,**D**) Western blot showing the expression and quantification of over-expressed KYAT1 enzyme after 48 h of transfection in HEPG2 cells. (1) control, (2) mock-transfected, and (3) KYAT1-over-expressing plasmid-transfected cells (*n* = 4). (**E**) Transamination activity (*n* = 10) and (**F**) Beta-elimination (*n* = 9–14) activity in control, mock, and KYAT1 over-expressed HEPG2 cell lysate. (**G**) KYAT1 over-expression increases the sensitivity of MSC-mediated cytotoxicity at 72 h in HEPG2 cells (*n* = 14). (**C**,**D**) KYAT1 48 kDa and Vinculin 124 kDa were used as a loading control and 20 µg of protein from cell lysate was used. (**E**–**G**) Graph represents mean ± SD, statistical analysis performed with one-way ANOVA with 95% confidential interval followed by Tukey’s multiple comparison test (ns = not significant, * *p* < 0.05, ** *p* < 0.01, *** *p* < 0.001, and **** *p* < 0.0001 compared with untransfected control and **^#^** *p* < 0.05, **^##^** *p* < 0.01, **^###^** *p* < 0.001, and ^####^ *p* < 0.0001 compared with mock).

MSC cytotoxicity was evaluated in the three HCC cell lines HEPG2, Hep3B, Huh7 cells and in freshly isolated primary human hepatocytes. Exposure of HCC cell lines and primary human hepatocytes to MSC resulted in IC_50_ values of 1152 ± 188, 1875 ± 171, 1294 ± 336, and 1218 ± 364 µM, respectively, indicating relatively similar cell sensitivities to MSC exposure in all the cell lines except for Hep3B cells (Figure 1A). The transamination activity was similar in HCC cell lines and primary hepatocytes. The calculated enzyme activity was 0.8 ± 0.3, 0.6 ± 0.2, 1.4 ± 0.3, and 0.8 ± 0.7 nmol of PPA-enol formed/min/mg of protein in HEPG2, Hep3B, Huh7, and primary hepatocytes, respectively (Figure 1B). The reduced sensitivity (IC50 value) measured in Hep3B correlated with reduced KYAT1 activity (Figure 1A,B). 

### 3.2. KYAT1 Plays a Major Role in MSC Metabolism in HCC Cell Lines

KYAT1 was overexpressed in HEPG2 cells using a plasmid that encoded the full-length enzyme. After 48 h post-transfection, overexpression was confirmed by Western blot (Figure 1C,D). Transamination (Figure 1E) and β-elimination (Figure 1F) activities were quantified in KYAT1 over-expressed cell lysate. Transamination activity (0.7 ± 0.3, 0.9 ± 0.2 and 11.6 ± 2.6 nmol of PPA-enol formed/min/mg of protein in un-transfected, mock and KYAT1 over-expressed cells, respectively) and β-elimination activity (0.4 ± 0.2, 0.4 ± 0.2 and 0.9 ± 0.3 µmol of NADPH consumed/min/mg of protein in un-transfected, mock and KYAT1 over-expressed cells, respectively) were significantly increased in KYAT1 over-expressed HEPG2 cells. KYAT1 overexpression sensitized HEPG2 cells to MSC treatment, resulting in significantly reduced IC_50_ values (316 ± 102 µM) as compared to non-transfected and mock-transfected controls (1013 ± 163 and 960 ± 198, respectively) (Figure 1G).

In contrast, siRNA-mediated knockdown provided a protective effect on MSC-mediated cytotoxicity in Huh7 cells (Supplementary Figure S1A,B). Overall, our results demonstrate that KYAT1-over-expressing cells displayed significantly higher transamination and β-elimination activities, combined with significantly increased cytotoxicity upon exposure to MSC. 

### 3.3. MSC Treatment Increased the ROS Production and Induced an Oxidative Environment Intracellularly in HCC Cells

**Figure 2:**
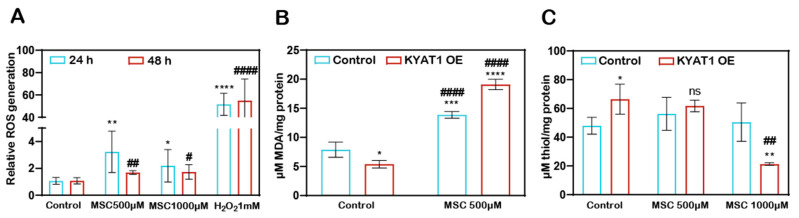
MSC-induced cytotoxicity by the induction of cytosolic and lipid ROS. (**A**) Cytosolic ROS production was assessed in Huh7 cells with MSC treatment over time (24 and 48 h) by the H_2_DCFDA-SRB method. About 1 mM of H_2_O_2_ was used as a positive control (*n* = 3). (**B**) Lipid ROS production was assessed in MSC-treated Huh7 cells with or without KYAT1 induction at 48 h by measuring the end product of lipid peroxidation i.e., malondialdehyde. (*n* = 3) (**C**) MSC-treated KYAT1 overexpressed Huh7 cells oxidizes the intracellular thiols at higher concentrations at 48 h (*n* = 3). (**A**–**C**) Graph represents mean ± SD, statistical analysis was performed with student t-test (ns = not significant, * *p* < 0.05, ** *p* < 0.01, *** *p* < 0.001, and **** *p* < 0.0001 compared with (**A**) 24 h control (**B**,**C**) mock-transfected control and **^#^** *p* < 0.05, **^##^** *p* < 0.01, and **^####^** *p* < 0.0001 compared with (**A**) 48 h control (**B**,**C**) KYAT1-overexpressed control).

MSC-induced ROS-dependent cytotoxicity to HCC cells. The H_2_DCFDA assay method showed that the Huh7 cells treated with MSC increased the production of ROS at 24 and 48 h (Figure 2A). KYAT1 induction in Huh7 cells increases the MSC metabolism, which resulted in increased lipid peroxidation activity significantly at 0.5 mM of MSC at 48 h (Figure 2B) and promotes an oxidative intracellular environment at 1 mM of MSC (Figure 2C). 

### 3.4. Intracellular Delivery of KYAT1mRNA Using Lipid Nanoparticles

**Figure 3:**
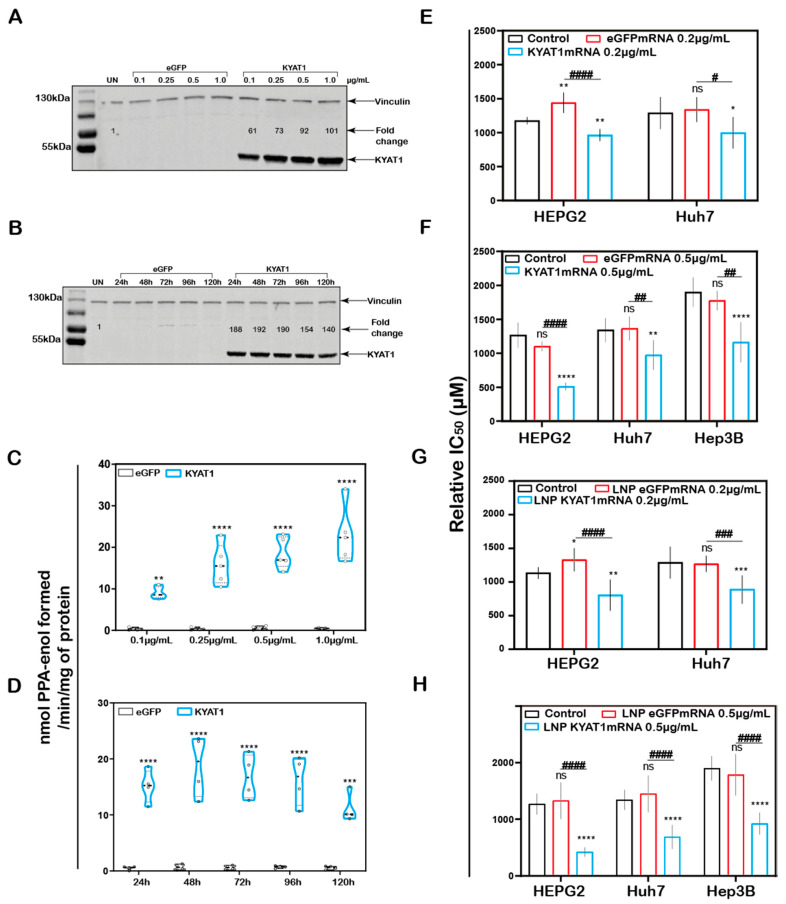
**KYAT1 upregulation increases the efficacy of MSC cytotoxicity in HCC cell lines.** Western blot showing the expression of KYAT1 in HEPG2 cells transfected with (**A**) varying concentrations (0.1, 0.25, 0.5, and 1 µg/mL) of KYAT1mRNA or eGFPmRNA encapsulated in LNP at 48 h. (**B**) About 0.5 µg/mL of LNP encapsuled KYAT1mRNA or eGFPmRNA at different time points (24, 48, 72, 96, and 120 h). (**C**) Transamination activity of KYAT1 was determined for samples at varying concentration at fixed time (*n* = 5) and (**D**) fixed concentration (0.5 µg/mL) at varying time (*n* = 4). **Dose-dependent effect of MSC at 72 h in HCC cell lines transfected with** (**E**) mRNA-encoding KYAT1 or eGFP (0.2 µg/mL), (*n* = 6–11) (**F**) mRNA-encoding KYAT1 or eGFP (0.5 µg/mL) (*n* = 5–11), (**G**) LNP-encapsulated KYAT1 or eGFP mRNA (0.2 µg/mL) (*n* = 6–11) and (**H**) LNP-encapsulated KYAT1 or eGFP mRNA (0.5 µg/mL) (*n* = 6–11). (**A**,**C**) KYAT1 48 KDa and Vinculin 124 KDa used as loading control, UN = un-transfected control. (**E**–**H**) eGFPmRNA and non-transfected cells were used as a control with respective concentrations as mentioned above. Graph represents mean ± SD, statistical analysis performed with (**B**,**D**) unpaired t-test and (**E**–**H**) one-way ANOVA with 95% confidential interval followed by Tukey’s multiple comparison test (ns = not significant, * *p* < 0.05, ** *p* < 0.01, *** *p* < 0.001, and **** *p* < 0.0001 compared with control and **^#^** *p* < 0.05, **^##^** *p* < 0.01, **^###^** *p* < 0.001, and **^####^** *p* < 0.0001 compared with eGFPmRNA).

In addition to the plasmid (DNA)-based over-expression system, we investigated the delivery of mRNA encoding for KYAT1, encapsulated in lipid nanoparticles (LNPs). HEPG2 cells were exposed to increasing concentrations of KYAT1-encoding mRNA-LNPs. The normalized signal intensity of Western blot revealed an increase in KYAT1 protein expression from the lowest concentration of 0.1 µg/mL (60-fold compared to control) to 1.0 µg/mL sample (100-fold compared to control) (Figure 3A). Protein determined over time in HEPG2 cells transfected with 0.5 µg/mL of KYAT1 mRNA, revealed 190-fold increased KYAT1 expression levels at 48 h compared to control, with a small and slow decline over time (Figure 3B). Enzyme activity assays correlated with the protein expression level (Figure 3C,D, Supplementary Table S1A,B), showing minimal differences in transamination activity in HEPG2 cells transfected with varying KYAT1mRNA concentrations at a fixed time point, and fixed concentration with varying time points. As a consequence of the proven over-expression of KYAT1 following efficient LNP mRNA delivery, we decided to limit our further exposed concentration to 0.2 and 0.5 µg/mL of mRNA.

### 3.5. Increased MSC Cytotoxicity in HCC Cell Lines upon KYAT1mRNA Delivery Using Lipid Nanoparticles or Lipofectamine

MSC cytotoxicity was measured in all three HCC cell lines following transfection with KYAT1 mRNA. Both mRNA concentrations (0.2 and 0.5 µg/mL) resulted in enhanced sensitivity to MSC cytotoxicity in all three HCC cell lines (Figure 3E–H). Unformulated KYAT1mRNA (0.2 µg/mL) sensitized HEPG2, Hep3B, and Huh7 cells to MSC significantly as compared to un-transfected or eGFPmRNA-transfected cells (Figure 3E, Table 1A). As hypothesized, increased KYAT1mRNA concentrations (0.5 µg/mL) further sensitized HEPG2, Hep3B, and Huh7 cells to MSC treatment (Figure 3F, Table 1A). Furthermore, cells transfected with the LNP-encapsulated KYAT1 showed a higher or similar sensitization effect toward MSC in the presence of 0.2 and 0.5 µg/mL of KYAT1mRNA (Figure 3G,H and Table 1B). Cells transfected with LNP-based mRNA delivery showed higher sensitivity towards MSC cytotoxicity as compared to lipofectamine-transfected cells (Table 1). Our experimental data also showed that LNP delivery per se did not affect MSC uptake and cytotoxicity in HCC cell lines (Supplementary Figure S1C).

### 3.6. Pharmacological Modulation of KYAT1 Activity

**Figure 4:**
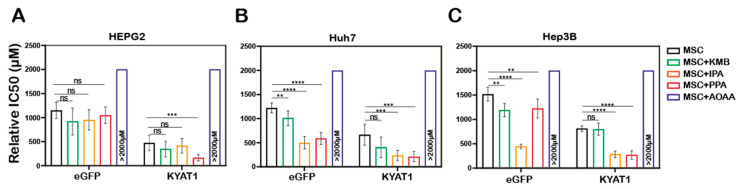
LNP-mediated KYAT1 over-expression in combination with α-ketoacids increased the efficacy of MSC treatment in HCC cell lines. Efficacy of MSC co-incubating with α-ketoacids such as phenylpyruvic acid (PPA) (400 µM), indole-3-pyruvic acid (IPA) (200 µM), α-keto-γ-(methylthio) butyric acid (KMB) (100 µM) and KYAT1 inhibitor aminooxy acetic acid (AOAA) (1 mM) in cells transfected with KYAT1mRNA encapsulated in LNP (0.5 µg/mL) in three different HCC cell lines at 72 h (**A**) HEPG2 (*n* = 8–10), (**B**) Hep3B (*n* = 6–8), and (**C**) Huh7 (*n* = 6–8). (**A**–**C**) LNPeGFPmRNA-transfected cells were used as a control with respective concentrations as above. Graph represents mean ± SD, statistical analysis performed with one-way ANOVA with 95% confidential interval followed by Tukey’s multiple comparison test (ns = not significant, ** *p* < 0.01, *** *p* < 0.001 and **** *p* < 0.0001 compared with control).

To modulate the β-elimination or transamination activity, several pharmacological-modulating compounds were tested and four different KYAT1 inducers and inhibitors were selected. Transamination and β-elimination activity assays were used to quantify KYAT1 activity in the presence of selected inducers and inhibitors in LNP-transfected cells (Supplementary Figure S2A–E). HEPG2, Hep3B, and Huh7 cells were transfected with 0.5 µg/mL eGFP or KYAT1mRNA encapsulated in LNP. The day after, transfected cells were exposed to MSC alone or in combination with inducers/inhibitors for an additional 72 h. Our results revealed that the KYAT1 inhibitor AOAA reversed MSC-induced cytotoxicity in all the cell lines and that 85% of cells persisted viable at 2 mM of MSC (Figure 4A–C).

In contrast, MSC combined with the α-ketoacid analog PPA (phenylpyruvic acid) significantly increased the cytotoxic effects in KYAT1-over-expressing cells, as compared to MSC alone in all three HCC cell lines (Figure 4A–C). Furthermore, the combination of MSC with IPA (indole-3-pyruvic acid) resulted in significant cytotoxic effects in KYAT1-over-expressing Huh7 and Hep3B cells compared with MSC alone (Figure 4B,C). Finally, KMB (α-keto-γ-(methylthio) butyric acid) in combination with MSC did not yield any significant additive effect (Figure 4A–C, Supplementary Table S2). KYAT1 inducers and inhibitors did not significantly alter MSC cytotoxicity in primary hepatocytes, except AOAA which protected MSC-induced cytotoxicity (Supplementary Figure S2F). In summary, the addition of α-ketoacid analogs in combination with MSC resulted in significantly increased cytotoxic effects only in HCC cell lines. 

### 3.7. Antisense microRNA Restricts MSC Induced Cytotoxicity to Tumor Cells 

**Figure 5:**
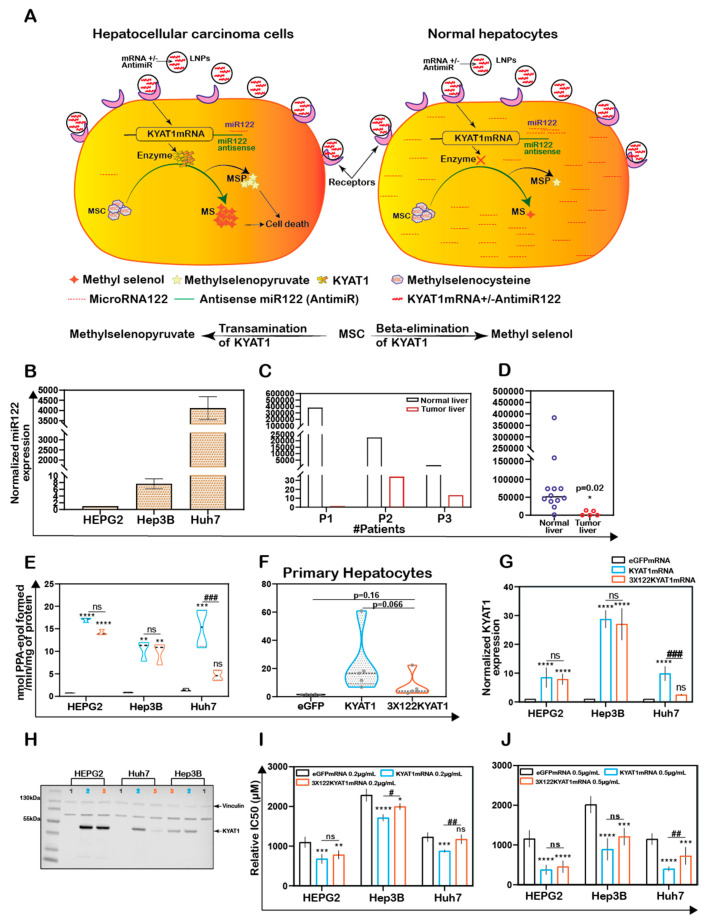
Endogenous expression of miRNA act as a targeted protein suppressor in cells transfected with mRNAs tagged with antisense miRNA target site. (**A**) Schematic representation of MicroRNA (miR122) assisted MSC metabolism via KYAT1 enzyme in cancer and normal hepatocytes. (**B**) Endogenous miR122 expression in HEPG2, Hep3B, and Huh7 cell lines by qRT-PCR (*n* = 4). (**C**) miR122 expression in human primary HCC compared with surrounding “normal” primary-hepatocytes from three different patients (*n* = 3). (**D**) Endogenous miR122 expression in freshly isolated healthy human hepatocytes (*n* = 12) and human primary HCC (*n* = 5). (**E**) Transamination activity of KYAT1 in cells transfected with eGFP, KYAT1, and 3 × miR122 KYAT1-over-expressing mRNA in HEPG2, Hep3B, and Huh7 cells (*n* = 3–4). (**F**) Transamination activity of KYAT1 in freshly isolated human hepatocytes transfected with eGFP, KYAT1, and 3 × miR122 KYAT1-over-expressing mRNA (*n* = 5). (**G**,**H**) Western blot showing the expression and quantification of KYAT1 in HEPG2 (*n* = 3), Hep3B (*n* = 3), and Huh7 (*n* = 4) cells 48 h after transfection with KYAT1mRNA and 3 × miR122 KYAT1mRNA. (**I**,**J**) MSC cytotoxicity in three different HCC cell lines (HEPG2, Hep3B, and Huh7) transfected with KYAT1 with or without incorporation of antisense miR122 target site. (**I**) 0.2 µg/mL and (**J**) 0.5 µg/mL of mRNA encoding eGFP, KYAT1 and 3 × miR122 KYAT1. (**B**–**D**) Since the HEPG2 cells have no or minimal expression of miR122, we used HEPG2 as a reference to calculate the miR122 expression in fold changes. Expression was normalized with miR192 and miR16. (**H**) KYAT1 48 KDa and Vinculin 124 KDa used as a loading control, (1) eGFP, (2) KYAT1, and (3) 3 × miR122 KYAT1. (**I**,**J**) eGFPmRNA was used as a control with respective concentrations as above (*n* = 5–7). Graph represents mean ± SD, statistical analysis performed with (**C**) unpaired t-test with Welch’s correction (**E**–**J**) one-way ANOVA with 95% confidential interval followed by Tukey’s multiple comparison test (ns = not significant, * *p* < 0.05, ** *p* < 0.01, *** *p* < 0.001, and **** *p* < 0.0001 compared with eGFP and ^#^ *p* < 0.05, ^##^ *p* < 0.01, ^###^ *p* < 0.001, and ^####^ *p* < 0.0001 compared with KYAT1).

To restrict over-expression of KYAT1 to only tumor cells, antisense sequences of specific microRNAs commonly downregulated in tumors were added in the 3′end of the coding region of KYAT1. Since selected microRNAs are tissue and disease-specific, we focused our attention on microRNA122 (miR122). A schematic representation of how miR122 may help us to target only HCC is presented in Figure 5A. Endogenous miR122 expression levels were initially quantified in human hepatocytes isolated from healthy donors and HCC, and compared with HEPG2, Hep3B, and Huh7 cell lines (Figure 5B–D). The calculated Ct values for HEPG2, Hep3B, and Huh7 cells were 32.7 ± 0.2, 29.4 ± 0.3, and 19.6 ± 0.1, and the corresponding fold changes were 1, 6.5 ± 2.5, and 4496 ± 887, respectively (Figure 5B). When miR122 level of expression was assessed in neoplastic tissue and compared with surrounding “normal” parenchyma, we measured 280,000-, 659-, and 81-fold increased miR122 expression in normal liver hepatocytes compared to neoplastic tissue in three different tested donors (Figure 5C). The calculated Ct value for freshly isolated human primary HCC (*n* = 5) and primary hepatocyte (normal) cells (*n* = 12) were 28.4 ± 3.7 and 21.4 ± 2.6, respectively, i.e., primary hepatocytes displayed a 17-fold higher miRNA122 expression compared to primary HCC (relative expression of HCC and normal hepatocytes were 5024 ± 6882 and 84,554 ± 101,817 respectively with *p*-value 0.02) (Figure 5C). Huh7 cells were characterized by elevated levels of miR122 expression compared to Hep3B (688-fold higher in Huh7) and HEPG2 (4496-fold higher in Huh7) cells. In this respect, Huh7 cell lines showed a comparable expression pattern to normal hepatocytes and thus we considered that they may serve as a relative control for normal hepatocytes. As a proof-of-concept, we delivered mRNA encoding for KYAT1 with three additional microRNA122 target sites (3 × 122 KYAT1mRNA) into HEPG2, Hep3B, Huh7, and freshly isolated primary hepatocytes. After 48 h, KYAT1 transamination activity was significantly reduced in Huh7 cells (Figure 5E) and freshly isolated primary hepatocytes (Figure 5F) transfected with 3 × 122 KYAT1mRNA as compared to KYAT1mRNA-transfected cells (Figure 5E,F). Indeed, Huh7 cells transfected with 3 × 122 KYAT1mRNA displayed a 70% reduction in protein level (Figure 5G,H). In contrast, Hep3B and HEPG2 cell lines did not display any significant reduction in KYAT1 activity (Figure 5G,H). 

HEPG2, Hep3B, and Huh7 cell lines were transfected with two different concentrations of KYAT1mRNA (0.2 and 0.5 µg/mL), with or without 3 × miR122 target sites. The relative IC_50_ values of MSC were measured at 72 h. A mild sensitization at 0.2 µg/mL of KYAT1 induction and significant sensitization at 0.5 µg/mL was observed with MSC in all three HCC cell lines (Figure 5I,J). Cells transfected with 0.2 µg/mL 3 × 122 KYAT1mRNA in Hep3B and Huh7 cells showed a significant protective effect against MSC cytotoxicity, which was supported by qRT-PCR analysis (see miR122 expression in Hep3B cells) (Figure 5B). At 0.2 µg/mL and 0.5 µg/mL concentrations, significant resistance towards MSC cytotoxicity was observed, limited to Huh7 cells (Figure 5I,J). The calculated relative IC_50_ values at 0.2 µg/mL and 0.5 µg/mL of eGFPmRNA, KYAT1mRNA, and 3 × 122KYAT1mRNA-transfected cells are presented in Table 2. 

Altogether, our results indicate that miRNA antisense targets are efficient in achieving tumor-specific cytotoxicity in cell types where miR122 is modulated.

### 3.8. Endogenous miRNA Can Protect Cells from MSC-Induced Cytotoxicity Even in Combination with KYAT1 Inducers/Inhibitors

HCC cell lines were transfected with two different concentrations of KYAT1mRNA (0.2 µg/mL and 0.5 µg/mL) with or without 3 × miR122 target sites. 

**Figure 6:**
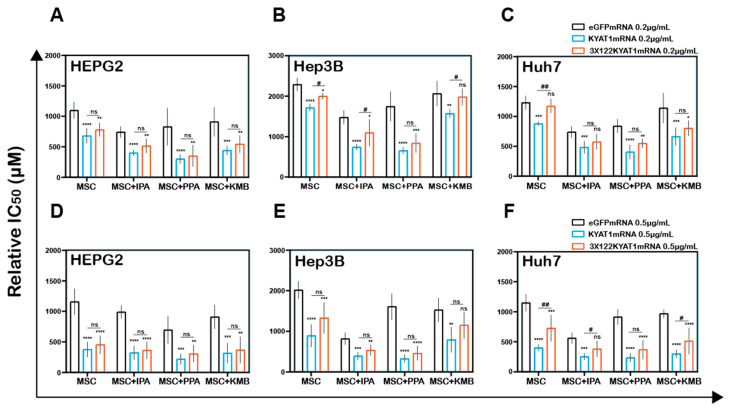
Antisense miR122 target site fusion with KYAT1 alleviates KYAT1 modifiers efficacy with MSC in KYAT1-overexpressed HCC cells. Effect of MSC co-incubated with modifiers in cells transfected with eGFPmRNA, KYAT1mRNA, and 3 × 122KYAT1mRNA. (**A**,**D**) HEPG2 cells (*n* = 5–7) (**B**,**E**) Hep3B cells (*n* = 5), and (**C**,**F**) Huh7 cells (*n* = 5–7). (**A**–**C**) About 0.2 µg/mL and (**D**–**F**) 0.5 µg/mL of mRNA encoding eGFPmRNA, KYAT1mRNA, and 3 × 122KYAT1mRNA. Modifiers such as phenylpyruvic acid (PPA) (400 µM), indole-3-pyruvic acid (IPA) (200 µM), and α-keto-γ-(methylthio) butyric acid (KMB) (100 µM) were co-incubated with MSC and relative IC_50_ values were calculated at 72 h. Graph represent mean ± SD, statistical analysis performed with one-way ANOVA with 95% confidential interval followed by Tukey’s multiple comparison test (ns = not significant, * *p* < 0.05, ** *p* < 0.01, *** *p* < 0.001, and **** *p* < 0.0001 compared with eGFP and ^#^ *p* < 0.05, ^##^ *p* < 0.01, ^###^ *p* < 0.001, and ^####^ *p* < 0.0001 compared with KYAT1).

HEPG2 cells transfected with KYAT1 containing 3 × miR122 did not show any protective effect with MSC when co-incubated with α-ketoacid (Figure 6A,D). Hep3B cells at low KYAT1 concentration (0.2 µg/mL) containing 3 × miR122 showed a significant protective effect in combined treatment with IPA and KMB, but these effects were diminished at the higher concentrations, 0.5 µg/mL (Figure 6B,E). Huh7 cells showed significant protective effects at 0.5 µg/mL KYAT1 concentration in combination with IPA and KMB. 

Interestingly we observed no significant differences in combined treatment with PPA at both 0.2 µg/mL and 0.5 µg/mL of KYAT1-transfected Huh7 cells (Figure 6C,F). Neither Hep3B nor Huh7 cells did show any significant protective effect when exposed to PPA. The calculated relative IC_50_ values are presented in Table 3A–C. Altogether, our data indicate that the off-target expression of KYAT1 protein can be controlled by endogenous microRNA in the presence of KYAT1 inducers/inhibitors except for PPA.

## 4. Discussions

Since Se-methylselenocysteine (MSC) is a pro-drug with very favorable pharmacokinetic properties and high per-oral bioavailability, the development of MSC-based strategies for the treatment of cancer is particularly relevant. Selenium-based chemotherapeutic regimens are not in clinical practice yet, due to the lack of systematic clinical trials and regimens that can provide a high degree of tumor specificity thereby limiting off-target effects. We present herein a novel strategy to achieve efficient and specific tumoricidal effects by combining the prodrug MSC with tumor-specific microRNA-guided overexpression of KYAT1 (Figure 5A). 

To study the endogenous KYAT1 activity we determined the half-maximal inhibitory concentration (IC_50_) in HCC cell lines in comparison with normal human hepatocytes, to establish a feasible study system of diverse responses generated by MSC treatment. Hep3B cells showed high resistance toward MSC cytotoxicity while HEPG2, Huh7, and normal primary cells showed similar IC_50_ values. Such differences in sensitivity to MSC treatment could be potentially explained by related KYAT1 activity [29]. To our knowledge, there have hitherto been limited reports on the roles of KYAT1 in cancer. KYAT1 plays an important role in tryptophan metabolism by metabolizing kynurenine to kynurenic acid (KYNA). KYNA has anti-proliferative activity attributed to its ability to inhibit p21, leading to cell cycle arrest [30]. Furthermore, KYNA exhibited clear anti-tumoral effects in human renal cell adenocarcinoma as well as in colon cancer cell models [31,32].

Overexpression of KYAT1 in HCC cell lines resulted in a significant alteration in the dose-response curve of MSC with a 65–70% decrease in IC_50_ values compared to empty vector or un-transfected cells. Our results confirm the preliminary analysis of the KYAT1-dependent cleavage of MSC [33]. To verify the increased enzymatic activity following KYAT1 over-expression, transamination and β-elimination activities were measured in cell lysates. Our results demonstrated a 15-fold higher transamination activity and a 2-fold higher β-elimination activity in KYAT1-overexpressed cell lysates. The sparse β-elimination activity is likely because KYAT1 favors transamination over β-elimination activity [33]. Methylselenol (β-elimination product of MSC) is known to induce ROS. Huh7 cells treated with MSC increased the production of cytosolic and lipid ROS. MSC at low concentration (250 µM in HEPG2 cells) showed a significant clearance in lipid ROS, but with KYAT1 induction we observed a significant increase in lipid ROS generation at the same dose of MSC, which might be due to higher MSC metabolism/methylselenol production by KYAT1 (Supplementary Figure S3). Furthermore, KYAT1 induction increased the rate of the reaction and elicited the oxidation of intracellular thiols resulting in oxidation of the intracellular milieu. Our data suggest that KYAT1-overexpressed cells suffer a prolonged induction of ROS when treated with MSC. This might result in constant cellular stress in HCC cells resulting in cell death.

The oxygen tension is an important factor in oxidative stress and the degree of ROS formation may be different during culture as compared to in vivo. The most common experimental condition is to cultivate cells under atmospheric oxygen. However, in atmospheric oxygen, the oxygen tension is 21% as compared to 13% as an average in vivo [34]. The oxygen tension may vary both in vitro and in vivo depending on tissues and cell types. Primary hepatocytes and HCC cell lines have a higher oxygen consumption rate (primary hepatocytes 200–400 amol/cell/s, HEPG2 110 amol/ng protein/s and Hep3B 160 amol/ng protein/s) [35,36]. These values suggest that primary hepatocytes and HCC cells grown in an incubator at 5% CO_2_ at 37 °C need a higher amount of oxygen compared to any other kind of cells. We cultured cells with the depth of 2–4 mm cell culture media in T25 (12 mL) or 35 mm plates (2 mL) or 96 well plate (100 µL per well) which results in 40–60 mmHg of pericellular oxygen concentration (PO_2_) [37]. Due to a higher oxygen consumption rate in hepatocytes, the rate of PO_2_ levels might decrease which may gradually result from normoxic to hypoxic conditions during this 72 h window. Since the cultivation of cells under atmospheric oxygen pressure with 5%CO_2_ is hyperoxic, the cells in our study model are likely subjected to a higher degree of oxidative stress as compared to in vivo conditions. 

Therefore, we were very meticulous in our ROS-measuring experiments i.e., we maintained the incubation time and media volume/height (to minimize the oxygen gradient changes), temperature (the reduction in temperature might cause oxidative stress to cells because of higher oxygen solubility). The seeding density of cells were carefully optimized before planning the experimental settings and cells were maintained about 85–95% confluent at the time of harvest. Our group has previously shown that ex vivo PDAC slice tissue cultured at 21% oxygen and 41% oxygen showed no differences in pimonidazole staining between normoxic and hyperoxic conditions [38]. We conclude that our data concerning ROS induction are also relevant in vitro even though the conditions used in the present study must be considered hyperoxic as compared to in vivo. The differences in oxygen tension should thus be considered in the evaluation of ROS-inducing drugs.

The use of mRNA is preferred over DNA to ectopically overexpress a protein since mRNA will only result in transient, controllable overexpression without risk of incorporation into the genome of the target cell, thus minimizing effects from transgene expression. Currently, RNA-based therapeutics are emerging in clinical studies in treating various diseases including cancer and various genetic disorders [39]. We employed an LNP-based delivery system to deliver KYAT1mRNA. Our results confirmed that (i) the LNP delivery system was an adequate and efficient tool for successful transfection, (ii) KYAT1 protein levels remained overexpressed for the duration of 120 h, and (iii) there was no interference of LNP particles with MSC cytotoxicity. 

Upon inducing KYAT1 via mRNA with (formulated mRNA) or without (unformulated mRNA) LNP encapsulation, the transfected HCC cell lines exhibited significantly increased sensitivity toward MSC compared to eGFPmRNA-transfected control. The KYAT1 activity depends on several factors, including the nucleophilicity of a substrate, PLP-PMP (co-factor) conversion rate and the presence of α-ketoacid [40,41]. The highly nucleophilic selenium moiety favors β-elimination over transamination which explains why MSC is a preferred substrate for KYAT1 compared to sulfur analogs [33]. To explore the impact of α-ketoacid analogs on the efficacy of KYAT-mediated MSC activation, several α-ketoacids were screened. We supplemented the culture medium with indole-3-pyruvic acid (IPA) [42], phenylpyruvic acid (PPA) [43] and α-keto-γ-(methylthio)butyric acid sodium salt (KMB) in the presence of MSC. The effect of IPA was unexpected given that the presence of this analog increased MSC cytotoxicity even without enzyme induction, indicating that the endogenous enzyme was activated by IPA. Han et al. showed that L-tryptophan and IPA were potent competitive endogenous inhibitors of KYAT1 [42]. They further investigated the possible mechanisms underlying the inhibition of IPA using indole-3-acetic acid (IAA) due to structural similarities between these two compounds and reported that IAA was not a substrate for KYAT1. It has been previously demonstrated that the moieties important for the binding within the active site were the indole ring and the carboxyl group [42]. This observation strongly suggests that MSC might be able to dock within the active site at the same time as IPA. Thus, the α-ketoacid analog in question may only inhibit the transaminase activity of KYAT1, promoting the production of cytotoxic MS. Unfortunately, IPA could not be used in the β-elimination activity assay because the color of this compound interfered with the β-elimination activity assay and showed a higher absorbance. On the other-hand, KYAT1 activity was significantly increased in the coupled-β-elimination activity assay in the presence of PPA and this explains the enhanced cytotoxicity with MSC [33]. Based on these results we conclude that sensitization is due to the self-renewal of PPA in cells. PPA reacts with L-tryptophan to generate L-phenylalanine which in turn reacts with 2-oxobutanoate to regenerate PPA. This continuous supply of PPA could thus represent an efficient pathway to continuously supply PLP required for the β-elimination reaction to proceed. It has previously been reported that the addition of phenylpyruvate to the transamination reaction abrogates the transamination activity of KYAT1 [33]. 

β-lyase inhibitors such as aminooxyacetic acid (AOAA) [44], and BFF-122 [45], were chosen to investigate its effect on MSC-mediated cytotoxicity/growth inhibitory effects. When the inhibitors were used in combination with MSC, AOAA protected the cells from MSC-mediated toxicity. AOAA is a nonspecific transamination inhibitor, previously shown to inhibit the activity of KYAT proteins [46]. A recent study presented a high-resolution crystal structure of the AOAA bound to PLP in the KYAT1 active site [47]. It was evident that the small molecule inhibitor irreversibly forms an oxime with the co-factor PLP that prevents its regeneration which in turn renders KYAT1 inactivation. This corroborates our finding of protection from MSC cytotoxicity when cells were co-treated with AOAA. MSC cleavage can occur by the enzyme CTH (cystathionase) which is a γ-lyase that can exert β-elimination depending on the substrate [29]. To rule out any involvement of endogenous cystathione gamma-lyase (γCTH) in the metabolism of MSC, both the γCTH inhibitor DL-propargylglycine (PAG) [48] and the substrate competitive inhibitor homoserine (HS) [49] were tested but did not show any significant efficacy in enzyme activity (Supplementary Figure S2).

Foster et al. showed an accumulation of Se in the pancreas, liver, and kidneys upon oral administration of ^75^Se [50]. Rooseboom et al. reported the highest level of β-elimination in the kidneys, followed by the liver, upon treatment with Se-cysteine conjugates [51]. Thus, the liver represents an ideal model to validate MSC as a prodrug, with the potential advantage of adding a high-level of tumor specificity by encapsulating target mRNA in LNPs coated with ligands that mediate entry via receptors on tumor cells. We employed a microRNA strategy to reduce off-target effects. Herein, we have demonstrated that the integration of the miR122 target site to mRNA reduced the off-target expression of KYAT1 i.e., the addition of multiple copies (3×) of a complementary miRNA122 site can suppress KYAT1 expression in cells that have a high level of endogenous miR122. miR122 is a phenotypic determinant of normal hepatocytes [21]. A similar miR122 approach was shown with apoptotic inducing proteins such as PUMA and caspase [20], but this is the first demonstration of modulation of a metabolic enzyme by this route. The use of a harmless metabolic enzyme is a great advantage compared to the induction of cell death proteins since any off-target effects would be less serious as compared to cell death proteins. This approach opens a new avenue to target and induce the expression of a protein of interest to designated transformed malignant cells and reduce off-target expression in normal cells.

MicroRNA-guided tumor-specific induction of KYAT1 in combination with the non-toxic prodrug (MSC) has a great potential in the treatment of cancers that are currently beyond cure such as HCC. A combination of factors controlling enzyme activity and tumor-specific delivery may offer further and improved therapeutic options. This concept could be extended to a variety of tumors by exploring cancer-specific ligands, antigens and miRNA combinations.

## Figures and Tables

**Table 1:** (**A**). Relative IC_50_ values of MSC at 72 h in HEPG2, Huh7 and Hep3B cells transfected with 0.2 µg/mL and 0.5 µg/mL of eGFPmRNA- or KYAT1mRNA using lipofectamine 3000. Results are presented as mean ± SD from triplicate measurements from at least five independent experiments. Resultant IC_50_ values are denoted in µM. ND = not determined. (**B**). Relative IC_50_ values of MSC at 72 h in HEPG2, Huh7, and Hep3B cells transfected with 0.2 µg/mL and 0.5 µg/mL of LNP encapsulated eGFPmRNA- or KYAT1mRNA. Results are presented as mean ± SD from triplicate measurements from at least five independent experiments. Resultant IC_50_ values are denoted in µM.

**HCC Cell Lines**	**Cells Transfected via Lipofectamine3000**				
	**Untransfected Cells**	**eGFPmRNA Control 0.2 µg/mL**	**KYAT1mRNA Control 0.2 µg/mL**	**eGFPmRNA Control 0.5 µg/mL**	**KYAT1mRNA Control 0.5 µg/mL**
HEPG2	1200 ± 157	1440 ± 151	964 ± 88	1101 ± 71	511 ± 55
Huh7	1315 ± 204	1339 ± 183	997 ± 232	1366 ± 176	973 ± 219
Hep3B	1900 ± 216	ND	ND	1776 ± 142	1164 ± 297
(**A**)					
**HCC Cell Lines**	**Cells Transfected via Lipid Nanoparticles (LNP)**				
	**Untransfected Cells**	**eGFPmRNA 0.2 µg/mL**	**KYAT1mRNA 0.2 µg/mL**	**eGFPmRNA 0.5 µg/mL**	**KYAT1mRNA 0.5 µg/mL**
HEPG2	1200 ± 157	1330 ± 171	804 ± 232	1327 ± 321	422 ± 80
Huh7	1315 ± 204	1270 ± 120	888 ± 211	1447 ± 326	644 ± 216
Hep3B	1900 ± 216	ND	ND	1883 ± 330	924 ± 191
(**B**)					

ND = not determined.

**Table 2:** Relative IC_50_ values of MSC at 72 h in HEPG2, Hep3B, and Huh7 cells transfected with 0.2 µg/mL and 0.5 µg/mL of eGFPmRNA, KYAT1mRNA, and 3 × 122KYAT1mRNA. Results are represented as mean ± SD from triplicate measurements from at least five independent experiments. Resultant IC_50_ values are denoted in µM.

Cell Lines	0.2 µg/mL of mRNA			0.5 µg/mL of mRNA		
	eGFP	KYAT1	3 × 122KYAT1	eGFP	KYAT1	3 × 122KYAT1
HEPG2	1100 ± 137	682 ± 121	781 ± 110	1159 ± 212	376 ± 123	456 ± 151
Hep3B	2288 ± 162	1717 ± 87	2000 ± 81	2018 ± 213	892 ± 278	1211 ± 217
Huh7	1230 ± 117	879 ± 36	1175 ± 119	1149 ± 144	400 ± 58	727 ± 221

**Table 3:** (**A**) Relative IC_50_ values for MSC alone or in combination with modifiers at 72 h in HEPG2 cells transfected with 0.2 µg/mL and 0.5 µg/mL of eGFPmRNA, KYAT1mRNA, and 3 × 122KYAT1mRNA. Results are represented as mean ± SD from triplicate measurements from at least five independent experiments. Resultant IC_50_ values are denoted in µM. (**B**) Relative IC_50_ values for MSC alone or in combination with modifiers at 72 h in Hep3B cells transfected with 0.2 µg/mL and 0.5 µg/mL of eGFPmRNA, KYAT1mRNA, and 3 × 122KYAT1mRNA. Results are represented as mean ± SD from triplicate measurements from at least five independent experiments. Resultant IC_50_ values are denoted in µM. (**C**) Relative IC_50_ values for MSC alone or in combination with modifiers at 72 h in Huh7 cells transfected with 0.2 µg/mL and 0.5 µg/mL of eGFPmRNA, KYAT1mRNA, and 3 × 122KYAT1mRNA. Results are represented as mean ± SD from triplicate measurements from at least five independent experiments. Resultant IC_50_ values are denoted in µM.

**Treatments**	**HEPG2 Relative IC50 in µM**					
	**0.2 µg/mL of mRNA**			**0.5 µg/mL of mRNA**		
	**eGFP**	**KYAT1**	**3 × 122 KYAT1**	**eGFP**	**KYAT1**	**3 × 122 KYAT1**
MSC	1100 ± 137	682 ± 121	781 ± 110	1159 ± 137	376 ± 123	456 ± 151
MSC + IPA	743 ± 89	401 ± 50	515 ± 116	990 ± 107	322 ± 116	364 ± 141
MSC + PPA	830 ± 305	301 ± 74	352 ± 180	697 ± 223	220 ± 88	309 ± 138
MSC + KMB	912 ± 243	442 ± 74	546 ± 144	911 ± 197	318 ± 165	369 ± 220
(**A**)						
**Treatments**	**Hep3B Relative IC50 in µM**					
	**0.2 µg/mL of mRNA**			**0.5 µg/mL of mRNA**		
	**eGFP**	**KYAT1**	**3 × 122 KYAT1**	**eGFP**	**KYAT1**	**3 × 122 KYAT1**
MSC	2288 ± 162	1717 ± 87	2000 ± 81	2018 ± 213	892 ± 278	1331 ± 384
MSC + IPA	1476 ± 169	743 ± 74	1097 ± 335	818 ± 150	392 ± 96	537 ± 133
MSC + PPA	1745 ± 365	652 ± 88	839 ± 237	1609 ± 321	328 ± 102	461 ± 181
MSC + KMB	2065 ± 312	1571 ± 102	1982 ± 93	1528 ± 296	793 ± 313	1152 ± 333
(**B**)						
**Treatments**	**Huh7 Relative IC50 in µM**					
	**0.2 µg/mL of mRNA**			**0.5 µg/mL of mRNA**		
	**eGFP**	**KYAT1**	**3 × 122 KYAT1**	**eGFP**	**KYAT1**	**3 × 122 KYAT1**
MSC	1230 ± 118	879 ± 36	1175 ± 119	1149 ± 144	400 ± 58	727 ± 221
MSC + IPA	741 ± 95	483 ± 103	575 ± 132	560 ± 93	254 ± 56	382 ± 131
MSC + PPA	840 ± 114	406 ± 121	550 ± 78	914 ± 131	236 ± 70	387 ± 158
MSC + KMB	1141 ± 253	663 ± 153	802 ± 128	969 ± 70	300 ± 68	514 ± 223
(**C**)						

## Data Availability

Data are available from the corresponding author upon request.

## Supplementary Materials

The following are available online at https://www.mdpi.com/article/10.3390/antiox10071094/s1. Figure S1A,B: KYAT1 knockdown protected the Huh7 cells from MSC-induced cytotoxicity. Figure S2A–F: KYAT 1 enzyme activity altered upon treatment with different pharmacological agents. Table S1A,B: Transamination activity of KYAT1 in HEPG2 cell lysate transfected with varying concentrations of eGFP and KYAT1mRNA encapsulated in LNP. Table S2: Relative IC_50_ values for MSC alone or in combination with modifiers at 72 h in HEPG2, Hep3B, and Huh7 cells transfected with 0.5 µg/mL of eGFPmRNA and KYAT1mRNA encapsulated in LNP.
