# Supplementary material for: A Novel mRNA-Mediated and MicroRNA-Guided Approach to Specifically Eradicate Drug-Resistant Hepatocellular Carcinoma Cell Lines by Se-Methylselenocysteine
Source: Antioxidants (Basel). 2021 Jul 7;10(7):1094. doi: 10.3390/antiox10071094 (PMC8301172; doi:10.3390/antiox10071094)
Supplement: Supplementary file 1 [file antioxidants-10-01094-s001.zip › antioxidants-1232517-supplementary.pdf]

Supplementary figures S1

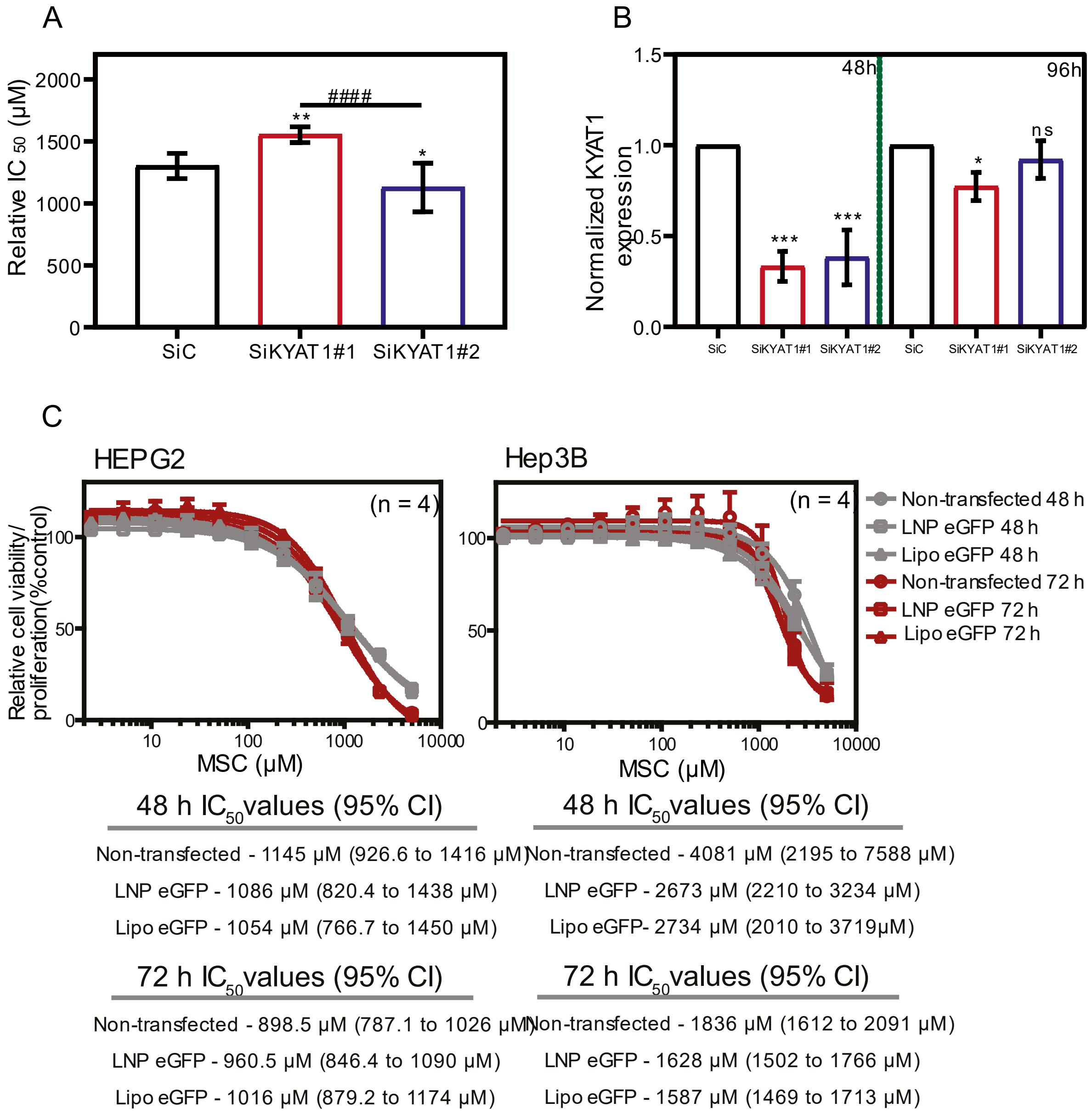

Supplementary figure S1. **KYAT1 knockdown protected the Huh7 cells from MSC induced cytotoxicity.** **(A)** KYAT1 siRNA mediated knockdown reverses the sensitivity of MSC mediated cytotoxicity at 72 h in Huh7 cells (n=8). The IC_{50} values of KYAT1 knockdown Huh7 cells were 1302±102, 1554±65 & 1128±196 for control siRNA, KYAT1 siRNA#1 & KYAT1 siRNA#2 respectively. **(B)** Verification of knockdown efficiency of two KYAT1 siRNA (Ambion, Life Technologies, Carlsbad, CA, USA, #S2498, #S225044) at 48 h & 96 h by qRT-PCR. Negative control siRNA (Ambion, Life Technologies, Carlsbad, CA, USA, #AM4611) was used as control and KYAT1 gene expression was normalized with HPRT1 gene (n=3). Graph represent mean ± SD, statistical analysis performed with one-way ANOVA with 95% confidential interval followed by **(A)** Tukey's multiple comparison test **(B)** Dunnett's multiple comparison test (ns = not significant, * $p < 0.05$, ** $p < 0.01$, *** $p < 0.001$ and **** $p < 0.0001$ compared with SiRNA control and $^{\#}$ p<0.05, $^{\#\#}$ p<0.01, $^{\#\#\#}$ p<0.001 & $^{\#\#\#\#}$ p<0.0001 compared between two KYAT1 SiRNA). **(C) Interference of LNP's with MSC cytotoxicity.** IC_{50} values of 0.5 µg/mL of eGFPmRNA (Lipo or LNP) transfected cells upon

MSC treatment in HEPG2 and Hep3b cells at 48 h (n=4) and 72 h (n=4) with 95% confidential intervals. Lipo eGFP: transfected with lipofectamine 3000 (Invitrogen).

Supplementary figures S2

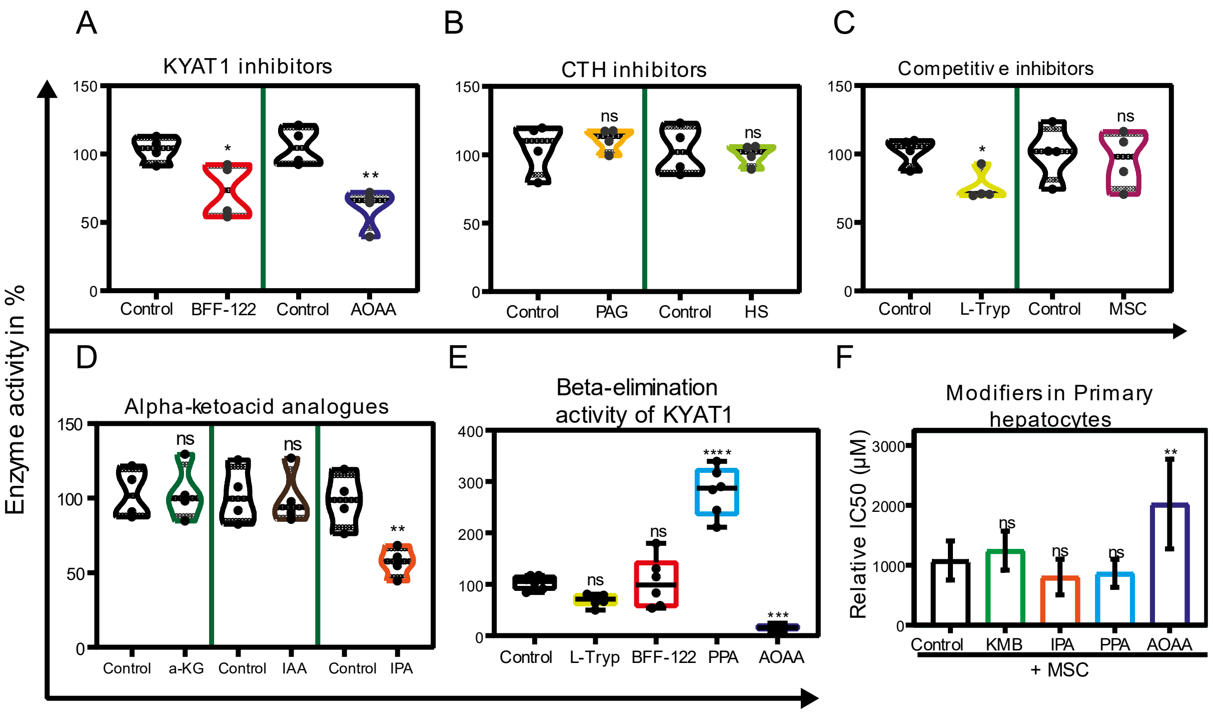

Supplementary figure S2. KYAT 1 enzyme activity altered upon treatment with different pharmacological agents. Transamination activity of KYAT1 was determined from cell lysates transfected with KYAT1mRNA encapsulated in LNP (0.5 μg/mL). **(A)** KYAT inhibitors (BFF-122, AOAA), (n=4). **(B)** Y-CTH inhibitors (PAG and HS), (n=4). **(C)** competitive inhibitors (L-Tryp and MSC) (n=4) and **(D)** α-Ketoacid analogs (α-KG, IAA and IPA) (n=4) relative to untreated control. **(E)** The same cell lysate was used to determine the beta-elimination activity of KYAT1 with different inhibitors and modifiers such as L-Try, BFF-122, PPA and AOAA, (n=6). **(F)** Cytotoxicity of MSC co-incubation with α-ketoacids/KYAT inhibitors (KMB, IPA, PPA and AOAA) in freshly isolated human hepatocytes at 72 h. IC_{50} values for MSC, MSC+KMB, MSC+IPA, MSC+PPA and MSC+AOAA were 1079± 325, 1243± 326, 803± 298, 867± 235 and 2021± 748 respectively (n=7). **(A-F)** 20μg of protein were used in transamination and beta-elimination activity assay along with different inhibitors and inducers such as L-Try, L-Tryptophan (2.0 mM); HS, Homoserine (400 μM); PAG, Propargylglycine (1.0 mM); IAA, 3-Indoleacetic acid (1.0 mM); BFF-122 (50 μM); IPA, Indole pyruvic acid (200 μM); PPA, phenylpyruvic acid (400μM) AOAA, Aminooxyacetic acid (1.0 mM); α-KG, α-Ketoglutarate/dimethyl 2-oxoglutarate (2.0 mM); MSC, Se-methylselenocysteine (5.0 mM) and KMB, Keto-γ-(methylthio) butyric acid (100μM). Graph represent mean ± SD, statistical analysis performed with **(A-D)** Unpaired t-test **(E)** **&** **(F)** one-way ANOVA with 95% confidential interval followed by Tukey's multiple comparison test (ns = not significant, * $p < 0.05$, ** $p < 0.01$, *** $p < 0.001$ and **** $p < 0.0001$ compared with untreated Control).

Supplementary figures S3

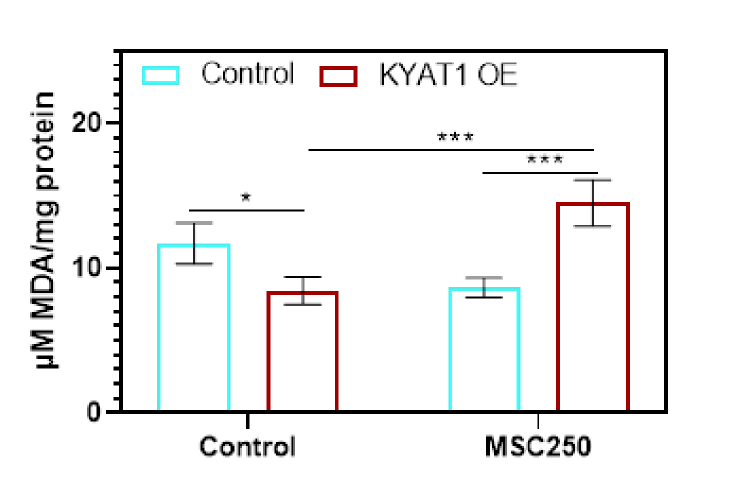

Supplementary figure S3. MSC induced Lipid ROS generation was measure at 48 h in HEPG2 cells induced with or without KYAT1 (0.5µg/mL), (n=3). Graph represent mean ± SD, statistical analysis was performed with 2way anova multiple comparisions test (ns = not significant, * $p < 0.05$, ** $p < 0.01$, *** $p < 0.001$ and **** $p < 0.0001$)

Supplementary tables S1

Table S1A. Transamination activity of KYAT1 in HEPG2 cell lysate transfected with varying concentrations of eGFP and KYAT1mRNA encapsulated in LNP. Results are presented as mean ± SD from at least five independent experiments.

Concentration in µg/mL	LNP eGFPmRNA	LNP KYAT1mRNA
	nmol of PPA-enol formed/min/mg of protein	
0.1	0.35±0.29	9.1±1.4
0.25	0.34±0.3	15.8±4.9
0.5	0.53±0.42	18.6±3.8
1.0	0.45±0.19	23±6.8

Table S1B. Transamination activity of KYAT1 in HEPG2 cell lysate measured at different time points in cells transfected with 0.5 µg/mL of eGFP and KYAT1mRNA encapsulated in LNP. Results are presented as mean ± SD from at least four independent experiments.

Time in hr	0.5µg/ml of LNP eGFPmRNA	0.5µg/ml of LNP KYAT1mRNA
	nmol of PPA-enol formed/min/mg of protein	
24	0.52±0.33	15.1±2.9
48	0.7±0.51	18.8±5.5
72	0.61±0.38	16.8±4.0
96	0.73±0.22	16.1±4.4
120	0.59±0.31	11.1±2.6

Supplementary tables S2

Table S2. Relative IC_{50} values for MSC alone or in combination with modifiers at 72 h in HEPG2, Hep3B and Huh7 cells transfected with 0.5 μg/mL of eGFPmRNA and KYAT1mRNA encapsulated in LNP. Results are presented as mean ± SD from triplicate measurements from at least six independent experiments. Resultant IC_{50} values are denoted in μM.

Treatments	HEPG2		Hep3B		Huh7	
	eGFP	KYAT1	eGFP	KYAT1	eGFP	KYAT1
MSC	1150±170	472±158	1517±138	810±58	1223±104	665±219
MSC+KMB	920±282	346±162	1190±139	797±126	1010±154	407±213
MSC+IPA	951±212	414±153	445±48	286±66	500±129	107±40
MSC+PPA	1051±169	165±67	1225±192	269±87	588±123	107±41
